# New Countermeasures Against Infections with/after COVID-19: Is Chlorine Dioxide a Useful and Safe Disinfectant?

**DOI:** 10.14789/jmj.JMJ22-0030-R

**Published:** 2022-10-15

**Authors:** KAORU OBINATA

**Affiliations:** 1 Department of Prevention Medicine for Mass Infection, Juntendo University Graduate School of Medicine, Tokyo, Japan; 1 Department of Prevention Medicine for Mass Infection, Juntendo University Graduate School of Medicine, Tokyo, Japan

**Keywords:** chlorine dioxide, COVID-19, aerosol infection, disinfection

## Abstract

Pandemics of microorganism are serious problem such as corona virus induced disease 2019(COVID-19), and the infectious diseases rapidly transmitted via airborne or aerosol among community space. To prevent aerosol infections, ozone and chlorine dioxide gases are practical methods in room air. However, ozone requires relatively high concentrations for this purpose, which might be toxic to humans present in the room. On the other hand, the low concentration of chlorine dioxide gas and aqueous solution are sufficiently effective against aerosol infection for the causative microorganism, and it is expected that when it is used in combination with a high-efficiency particulate air filter, it will be highly safe with high prevention effect and cost effectiveness.

## Introduction

Since the current global outbreak of severe acute respiratory syndrome coronavirus 2 (SARS-CoV-2) infection, the development of effective and safe methods to inactivate viruses in a manned environment have been required^1)^.

Respiratory viruses mainly transmitted human-to-human via droplets or aerosols. To control infections, not only standard precaution but also countermeasures at the spatial environment are important, and the establishment of economically healthy and effective infection prevention methods is desired.

## Disinfection methods

### Ozone

Ozone gas is a strong oxidant^2)^ and has long been used to inactivate pathogenic microbes in water^3, 4)^. It can be used to inactivate microbes on the surfaces of objects and aerosols. Hudson et al. found ozone inactivated viruses, including mouse coronavirus, on glass and stainless steel^5)^. They reported that 20-25 ppmv ozone with 90% relative humidity was effective at inactivating 12 viruses on hard or porous surfaces^5)^.

However, high concentrations of ozone need for the disinfection of room air or object surfaces, and problem remains its toxicity to humans and animals^6)^. Sokolowska et al. reported a mouse experiment where a single exposure to 1 ppmv ozone for 60 min caused damage to the bronchiolar epithelium within 2 hours, disrupted epithelial tight junctions, and promoted cell death, which was followed by reactive oxygen species production^6)^. This result indicates the difficulty of using ozone as a disinfectant against viruses in the presence of humans in rooms. Chronic exposure of humans to ozone causes the progressive and irreversible loss of alveolar epithelial cells and eventually emphysema occurs^7)^. Therefore, although the effectiveness of ozone has been proven, its use, especially in terms of concentrations and exposure periods, should be carefully controlled to avoid adverse effects.

### Chlorine dioxide

Chlorine dioxide inactivates many viruses. For instance, the inactivation activity of chlorine dioxide against feline calicivirus and influenza A virus was 0.05 ppmv for 5 h, and the reduction of virus was to a level of 10^-5^ ^8)^. Inactivation of viruses by chlorine dioxide aqueous solution was also demonstrated. Sanekata et al. found that human influenza virus, measles virus, canine distemper virus, human herpes virus, human and canine adenoviruses, and canine parvovirus were inactivated by chlorine dioxide aqueous solution^9)^. Hepatitis A virus was completely inactivated after 10 min in a 7.5 mg/L aqueous solution of chlorine dioxide^10)^. The cause of inactivation of the virus was related to the complete loss of antigenicity of the virus and the loss of the 5’ non-translated region of its genome^10, 11)^. Chlorine dioxide is a relatively stable free radical^12, 13)^ that denatures proteins by oxidizing their tyrosine and tryptophan residues^14-16)^. Ogata et al demonstrated that chlorine dioxide is effective against the SARS- CoV-2 and the mechanisms of action that inhibits the binding of the spike protein of the SARS-CoV-2 on ACE2 through the action of chlorine dioxide has been verified^17)^.

Akamatsu et al. demonstrated the safety of a low concentration of chlorine dioxide in an animal experiment. Rats exposed to whole-body inhalation of 0.1 ppmv chlorine dioxide for six months with a two-week recovery period showed no differences in body weight gain, food intake, water intake, relative organ weight, blood biochemistry data, and hematology examination data compared with control rats not exposed to chlorine dioxide^18)^. In their experiment, rats were exposed to chlorine dioxide for 24 hours/day and 7 days/week. Furthermore, the concentration of gas was precisely controlled within ± 25% of the target concentration^18)^. Their result strongly suggests that chlorine dioxide at or below 0.1 ppmv can be used safely to disinfect room air in the presence of humans for a long period. A concentration of 0.1 ppmv chlorine dioxide was effective at inactivating virus in room air^19-21)^. The US Department of Labor of the Occupational Safety and Health Administration (OSHA) stated that the permissible exposure concentration of chlorine dioxide for humans for an 8-hour time-weighted average was 0.1 ppmv^22)^. Dalhamn reported a no-observed adverse-effect-level (NOAEL) of 0.1 ppmv in rats exposed to chlorine dioxide for 5 hours/day for 10 weeks^23)^. Therefore, chlorine dioxide can be used effectively and safely at relatively low concentrations against viruses and bacteria.

### Ultraviolet (UV)

UV light and photochemical reactions have been used to inactivate viruses. Regarding UV light irradiation, 254 nm light is usually used^24, 25)^. While this method is quite useful for inactivating viruses floating in room air or stuck on objects in a room, its disadvantages include that it cannot inactivate virus in the blind spots of a room where UV light does not penetrate. Furthermore, humans cannot be present because UV irradiation causes cataracts^26)^ and dermal neoplasms^27)^. There are other similar methods that can inactivate viruses. For instance, viruses are inactivated by photochemical reactions using titanium oxide^28)^. However, this method is useful predominantly to inactivate viruses stuck on objects. Furthermore, it has not been proven to be effective at inactivating virus floating in room air or away from object surfaces.

### Hypochlorous acid (HClO)

Aerosol sprays are also used to disinfect room air containing pathological viruses. For this purpose, hypochlorous acid in water is frequently used as a spray to inactivate avian influenza virus, Newcastle disease virus, and coronavirus^29, 30)^. An aqueous solution of sodium hypochlorite (NaClO) was also used as a spray to disinfect viruses^31, 32)^. However, both hypochlorous acid and sodium hypochlorite solution sprays are primarily used to disinfect viruses on the surfaces of objects. While useful, they are rarely used to disinfect room air, and their effectiveness at inactivating viruses floating in room air has not been demonstrated quantitatively.

### Ventilation

The mechanical or natural ventilation of room air is a simple, effective, and inexpensive way to minimize the airborne transmission of respiratory viruses^33)^. The Center for Disease Control and Prevention recommends a ventilation of 6-15 room air changes per hour to minimize the transmission of microbes^33)^. However, the efficient ventilation of room air is accompanied by unwanted warming or cooling of room air unless an ambient temperature is appropriate. This requires extra energy expenditure aside from that required for mechanical ventilation, and such procedures go against the earth-warming policies of many countries. Thus, the safe and effective inactivation of viruses in room air by methods other than ventilation should consider energy saving. In summary, after a survey of the current literature, low-concentration chlorine dioxide is the most suitable agent for the safe and effective.

## Discussion

Ozone gas is an effective disinfectant in the air. However, its toxicity becomes a serious problem when it is used continuously in a room^5-7)^. On the other hand, chlorine dioxide gas can be used in a room with low concentrations that are effective in inactivating viruses and which are safe to humans^18)^. Chlorine dioxide gas is the only practical and currently available disinfection agent that can disinfect viruses in room air to prevent viral respiratory diseases^34)^.

**Figure 1:**
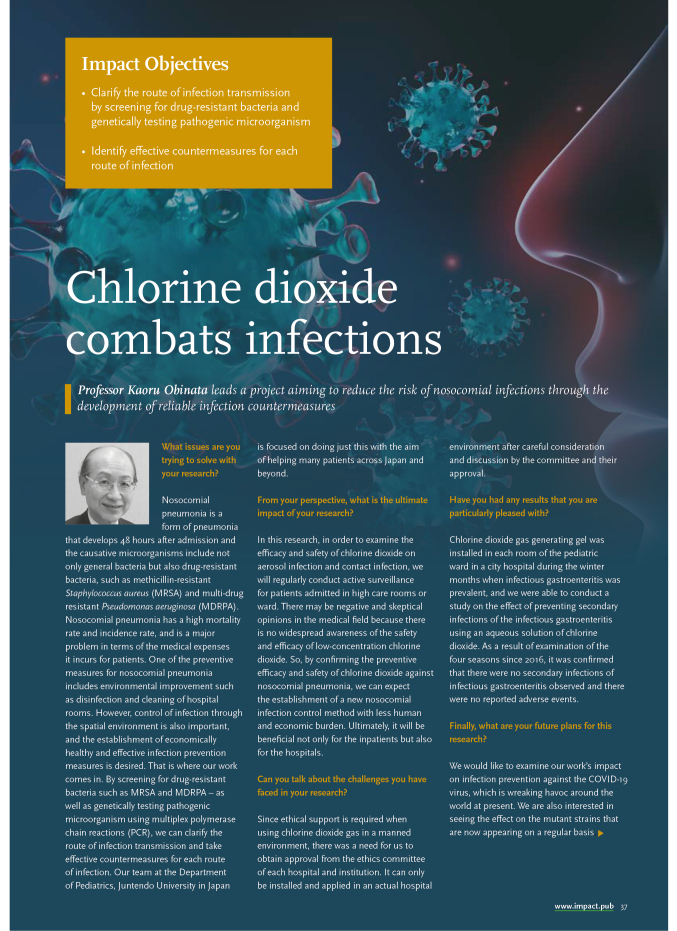
Chlorine dioxide combats infections

Since ethical support is required when using chlorine dioxide gas in a manned environment, there was a need to obtain approval from the ethics committee of hospital and institution. We installed and applied in an actual hospital environment after careful consideration and discussion by the committee and their approval. After that chlorine dioxide gas generating gel was installed in each room of the pediatric ward in a city hospital during the winter months when infectious gastroenteritis was prevalent, and we were able to conduct a study on the effect of preventing secondary infections of the infectious gastroenteritis using an aqueous solution of chlorine dioxide. As a result of examination of the four seasons since 2016, it was confirmed that there were no secondary infections of infectious gastroenteritis observed and that there were no reported adverse events. (Figure 1)

Nosocomial pneumonia develops in patient around 48 hours after admission. This form of pneumonia has a high mortality rate and incidence rate and is a major problem in terms of the medical expenses it incurs for patients. One of the preventive measures for nosocomial pneumonia includes environmental improvement such as disinfection and cleaning of hospital rooms. However, in many ways, this is an uphill battle, as many infections are transmitted through droplets in the air (as we have seen with the recent COVID-19 pandemic), which are extremely difficult to combat, especially when the main preventive method is cleaning surfaces.

**Figure 2:**
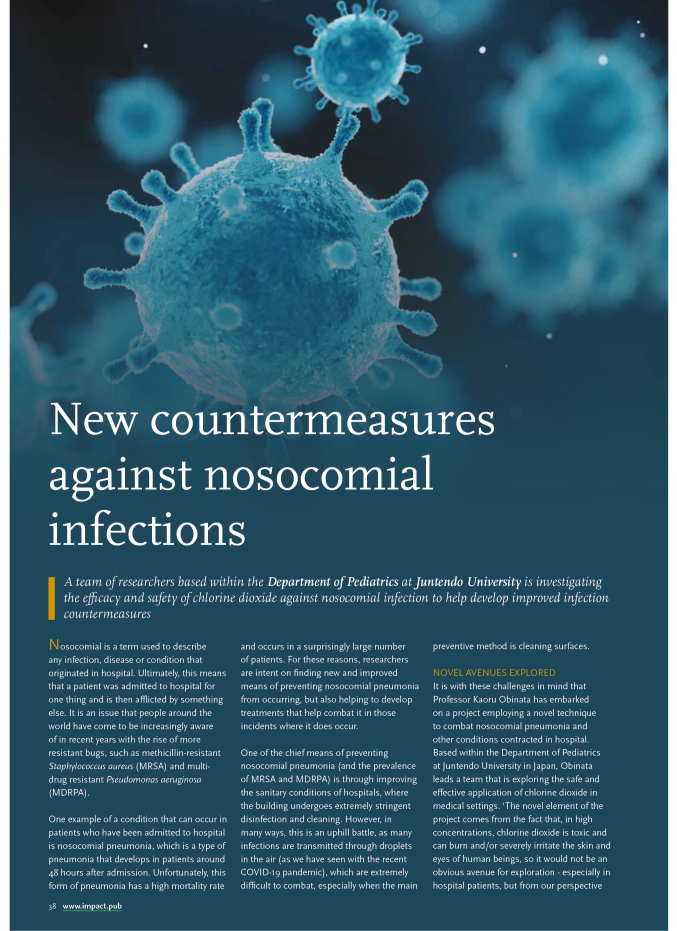

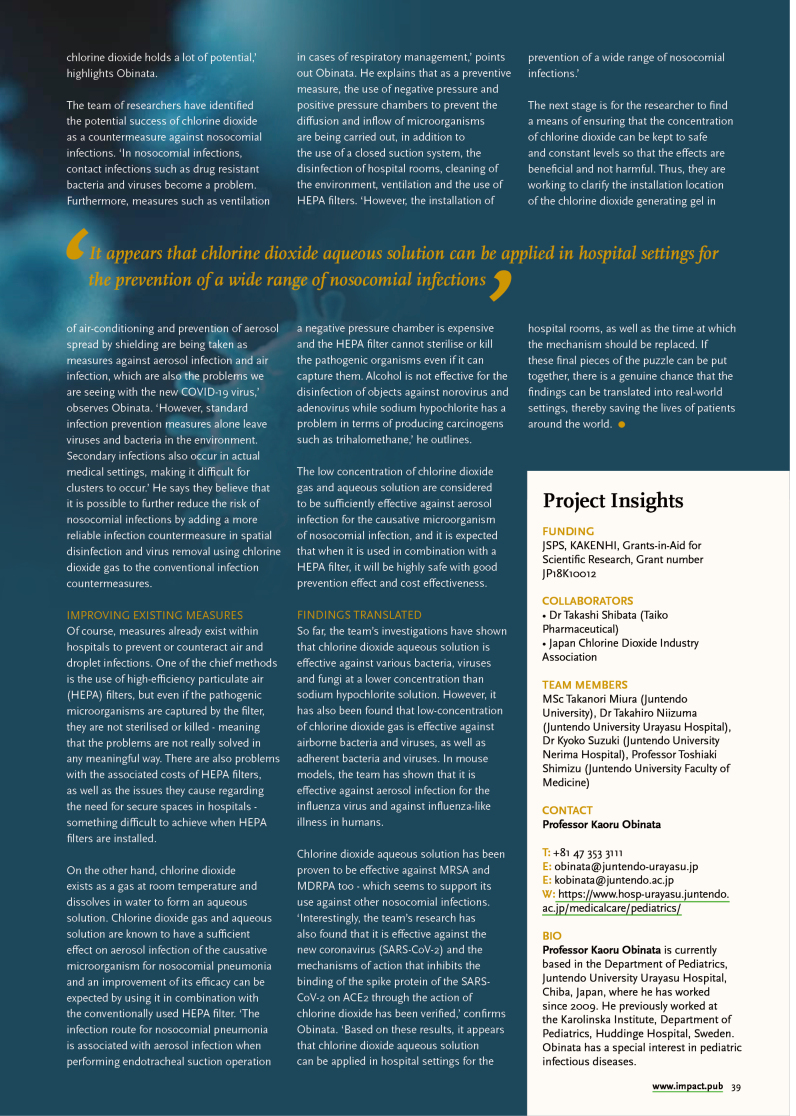
New countermeasures against nosocomial infection

In nosocomial infections, contact infections such as drug resistant bacteria and viruses become a problem. Furthermore, measures such as ventilation of air-conditioning and prevention of aerosol spread by shielding are being taken as measures against aerosol infection and air infection. Standard precaution measures alone leave viruses and bacteria in the environment. Secondary infections also occur in actual medical settings, making it difficult for clusters to occur. It is possible to further reduce the risk of nosocomial infections by adding more reliable infection countermeasure in spatial disinfection and virus removal using chlorine dioxide gas to the conventional infection countermeasures. And the causative microorganisms include not only general bacteria but also drug-resistant bacteria, such as Methicillin-Resistant *Staphylococcus aureus* (MRSA) and Multi-Drug Resistant *Pseudomonas aeruginosa* (MDRPA). (Figure 2)

Of course, measures already exist within hospitals to prevent or counteract air and droplet infections. One of the chief methods is the use of high-efficiency particulate air (HEPA) filters, but even if the pathogenic microorganisms are captured by the filter, they are not sterilized or killed - meaning that the problems are not really solved in any meaningful way. There are also problems with the associated costs of HEPA filters, as well as the issues they cause regarding the need for secure spaces in hospitals - something difficult to achieve when HEPA filters are installed.

On the other hand, chlorine dioxide exists as a gas at room temperature and dissolves in water to form an aqueous solution. Chlorine dioxide gas and aqueous solution have a sufficient effect on aerosol infection of the causative microorganism for nosocomial pneumonia and an improvement of its efficacy can be expected by using it in combination with the conventionally used HEPA filter.

The infection route for nosocomial pneumonia is associated with aerosol infection when performing endotracheal suction operation in cases of respiratory management, as a preventive measure the use of negative pressure and positive pressure chambers to prevent the diffusion and inflow of microorganisms are being carried out, in addition to the use of a closed suction system, the disinfection of hospital rooms, cleaning of the environment, ventilation and the use of HEPA filters. However, the installation of a negative pressure chamber is expensive, and the HEPA filter cannot sterilize or kill the pathogenic organisms even if it can capture them. Alcohol is not effective for the disinfection of objects against norovirus and adenovirus while sodium hypochlorite has a problem in terms of producing carcinogens such as trihalomethane.

The low concentration of chlorine dioxide gas and aqueous solution are sufficiently effective against aerosol infection for the causative microorganism of nosocomial infection, and it is expected that when it is used in combination with a HEPA filter, it will be highly safe with high prevention effect and cost effectiveness.

Chlorine dioxide aqueous solution has been proven to be effective against MRSA and MDRPA, which seems to support its use against other nosocomial infections. Based on these results, it appears that chlorine dioxide aqueous solution can be applied in hospital settings for the prevention of a wide range of nosocomial infections. The next stage is to find a means of ensuring that the concentration of chlorine dioxide can be kept to safe and constant levels so that the effects are beneficial and not harmful. Thus, we will work to clarify the installation location of the chlorine dioxide generating gel in hospital rooms, as well as the time at which the mechanism should be replaced^35)^ (Figure 2).

If these final pieces of the puzzle can be put together, there is a genuine chance that the findings can be translated into real-world settings, thereby saving the lives of patients around the world.

## Funding

Grants-in-Aid for Scientific Research (KAKENHI) JSPS Grant Number JP 18K10012.

## Author contributions

KO. wrote the manuscript.

## Conflicts of interest statement

The author declares that there are no conflicts of interest.
